# Novel germline mutations and unclassified variants of *BRCA1* and *BRCA2* genes in Chinese women with familial breast/ovarian cancer

**DOI:** 10.1186/s12885-016-2107-6

**Published:** 2016-02-06

**Authors:** Wen-Ming Cao, Yun Gao, Hong-Jian Yang, Shang-Nao Xie, Xiao-Wen Ding, Zhi-Wen Pan, Wei-Wu Ye, Xiao-Jia Wang

**Affiliations:** Department of Medical Oncology, Zhejiang Cancer Hospital, 38 Guangji Road, Hangzhou, 310022 China; Institute of Cancer Research, Zhejiang Cancer Hospital, Hangzhou, 310022 China; Department of Breast Cancer Surgery, Zhejiang Cancer Hospital, Hangzhou, 310022 China; Department of Clinical Laboratory, Zhejiang Cancer Hospital, Hangzhou, 310022 China

**Keywords:** *BRCA1*, *BRCA2*, Germline mutation, Unclassified variants, Founder mutation, Chinese women

## Abstract

**Background:**

Germline mutations in the *BRCA1* and *BRCA2* genes greatly increase a woman’s risk of developing breast and/or ovarian cancer. The prevalence and distribution of such mutations differ across races/ethnicities. Several studies have investigated Chinese women with high-risk breast cancer, but the full spectrum of the mutations in these two genes remains unclear.

**Methods:**

In this study, 133 unrelated Chinese women with familial breast/ovarian cancer living in Zhejiang, eastern China, were enrolled between the years 2008 and 2014. The complete coding regions and exon-intron boundaries of *BRCA1* and *BRCA2* were screened by PCR-sequencing assay. Haplotype analysis was performed to confirm *BRCA1* and *BRCA2* founder mutations. In silico predictions were performed to identify the non-synonymous amino acid changes that were likely to disrupt the functions of *BRCA1* and *BRCA2*.

**Results:**

A total of 23 deleterious mutations were detected in the two genes in 31 familial breast/ovarian cancer patients with a total mutation frequency of 23.3 % (31/133). The highest frequency of 50.0 % (8/16) was found in breast cancer patients with a history of ovarian cancer. The frequencies of *BRCA1* and *BRCA2* mutations were 13.5 % (18/133) and 9.8 % (13/133), respectively. We identified five novel deleterious mutations (c.3295delC, c.3780_3781delAG, c.4063_4066delAATC, c.5161 > T and c.5173insA) in *BRCA1* and seven (c.1-40delGA, c.4487delC, c.469_473delAAGTC, c.5495delC, c.6141T > A, c.6359C > G and c.7588C > T) in *BRCA2*, which accounted for 52.2 % (12/23) of the total mutations. Six recurrent mutations were found, including four (c.3780_3781delAG, c.5154G > A, c.5468-1del8 and c.5470_5477del8) in *BRCA1* and two (c.3109C > T and c.5682C > G) in *BRCA2*. Two recurrent *BRCA1* mutations (c.5154G > A and c.5468-1del8) were identified as putative founder mutations. We also found 11 unclassified variants, and nine of these are novel. The possibility was that each of the non-synonymous amino acid changes would disrupt the function of *BRCA1* and *BRCA2* varied according to the different algorithms used.

**Conclusions:**

*BRCA1* and *BRCA2* mutations accounted for a considerable proportion of hereditary breast/ovarian cancer patients from eastern China and the spectrum of the mutations of these two genes exhibited some unique features. The two *BRCA1* putative founder mutations may provide a cost-effective option to screen Chinese population, while founder effects of the two mutations should be investigated in a lager sample size of patients.

## Background

In 2009, the morbidity rate of breast cancer was 42.55 *per* 100,000 Chinese women, and breast cancer ranked first in cancer incidence and fifth in cancer-related deaths among females [1]. The mean age at diagnosis of breast cancer is 45–55 years in Chinese women, which is considerably younger than that in western women [2]. A significant proportion of breast cancer in Chinese women is caused by genetic alterations. Germline mutations in many genes, such as *BRCA1*, *BRCA2*, *ATM*, *TP53*, *RAD51C and XRCC2*, have been identified to be associated with breast cancer [3–5]. Several studies have investigated germline mutations in genes including *BRCA1*, *BRCA2*, *TP53*, *BRIP1*, *PALB2*, *CHEK2*, *RAD50*, *NBS1* and *RAD51C* in Chinese women with high risk breast cancer [6–21]. We previously summarized the spectrum of the germline mutations in these genes and found that the *BRCA1* and *BRCA2* tumor suppressor genes are the two most important susceptibility genes and account for nearly 98 % of hereditary breast cancer in China [22]. We found that the spectrum of *BRCA1* and *BRCA2* germline mutations in Chinese high risk breast cancer patients are much smaller than those in Caucasian patients, and little has been recognized in this field. The overall mutation frequencies in these two genes in Chinese high risk breast cancer patients ranged from 8.3 to 27.8 %, depending on the detection methods and patient inclusion criteria used. These frequencies are much lower than the 25–40 % in *BRCA1* and 6–15 % in *BRCA2* that have been observed in Caucasian populations [22]. Because germline mutations in *BRCA1* and *BRCA2* greatly increase a woman’s risk of developing breast and/or ovarian cancer, and the prevalence and distribution of the germline mutations differ in different races/ethnicities, we were interested in identifying the full spectrum of these mutations in high-risk female breast cancer patients in the Chinese population.

In this study, we screened the entire coding regions and exon-intron boundaries of the *BRCA1* and *BRCA2* genes in 133 familial breast/ovarian cancer patients from eastern China. A total of 23 deleterious mutations, including 12 novel mutations (five in *BRCA1* and seven in *BRCA1*), were detected in these two genes in 31 familial breast/ovarian cancer patients, and the total mutation frequency was 23.3 % (31/133). The highest frequency of 50.0 % (8/16) was found in the breast cancer patients with a history of ovarian cancer. Six recurrent mutations were found, including four in *BRCA1* and two in *BRCA2*. We also found 11 unclassified variants (UVs), nine of which were novel. Additionally, using comparative evolutionary bioinformatic programs, we identified the non-synonymous amino acid changes that are likely to disrupt the functions of the *BRCA1* and *BRCA2* genes. Our study suggested that *BRCA1* and *BRCA2* mutations accounted for a considerable proportion of the hereditary breast/ovarian cancer patients in eastern China and that the spectrum of the mutations in these genes exhibited unique features.

## Methods

### Subjects

All patients were diagnosed between 2008 and 2014 in the Zhejiang Cancer Hospital, eastern China. The criterion for familial breast/ovarian cancer was that at least one first- or second-degree relative of the breast cancer patient had been affected by breast cancer and/or ovarian cancer, regardless of age. Written consent was obtained from all participating patients. The study was approved by the Research and Ethics Committee of Zhejiang Cancer Hospital, China. Peripheral blood samples were drawn from at least one affected person in each family and stored in EDTA tubes at−80 °C. A total of 133 patients from unrelated families were enrolled in this study. For the 62 patients who enrolled before 2012, the *BRCA1* gene was analyzed with a polymerase chain reaction (PCR)-sequencing assay as previously reported [13], and the mutations of the *BRCA2* gene were screened in this study.

### *BRCA1* and *BRCA2* mutation analysis

Genomic DNA was extracted from the peripheral blood leukocytes of one patient from each family using a ZR Genomic DNA Kit (Zymo Research, Orange County, CA, USA) or a QIAamp DNA Blood Mini kit (Qiagen, Hilden, Germany). The entire coding regions and exon-intron boundaries of *BRCA1* [U14680.1] and *BRCA2* [U43746.1] were screened using PCR-sequencing assay. Totals of 32 pairs and 40 pairs of primers for *BRCA1* and *BRCA2*, respectively, were synthesized by Invitrogen. The primers and PCR conditions are available on request. The PCR products were verified on standard agarose gels prior to mutation analysis and purified by membrane retention. The purified fragments were sequenced using a BigDye Terminator Cycle Sequencing Kit and an ABI 3130xl Genetic Analyzer (Applied Biosystems, Foster City, CA, USA). All mutations were confirmed by duplicate independent PCR. No screening for large genomic rearrangements was performed.

All of the mutations and variants were named according to the Human Genome Variation Sequence systematic nomenclature (HGVS; http://www.hgvs.org/mutnomen/). The Breast Cancer Information Core (BIC) nomenclature (https://research.nhgri.nih.gov/projects/bic/Member/index.shtml) was also indicated in the tables and text because this system had been widely employed in many studies. All of the mutations and variants were queried against the 1000 Genomes database using the 1000 Genomes Browser (http://browser.1000genomes.org/) to determine whether the mutations and variants had been reported in the Chinese population.

### Haplotype analysis

Haplotype analysis was conducted on the unrelated patients with recurrent *BRCA1* or *BRCA2* germline deleterious mutations. Thirteen microsatellite polymorphic loci were used (*BRCA1* D17S855, D17S1322, D17S1323, D17S1326, D17S1327; *BRCA2* D13S1304, D13S217, D13S289, D13S1699, D13S1698, D13S171, D13S1695, D13S267) [9, 12]. Primer sequences of all microsatellite polymorphic loci were obtained from the Probe Database (http://www.ncbi.nlm.nih.gov/probe). PCR products fluorescently labeled were size fractioned on an ABI 3730xl Analyzer (Applied Biosystems) using GeneScan 500 LIZ Size Standard. Analysis was performed using the Genemarker v1.5 analysis software.

### In silico prediction

To identify the UVs that were likely to disrupt the functions of the *BRCA1* and *BRCA2* genes, we performed in silico predictions with the following six comparative evolutionary bioinformatic programs: Align-GVGD (http://agvgd.iarc.fr/agvgd_input.php), SIFT (http://sift.jcvi.org/), PROVEAN (http://provean.jcvi.org/index.php), PolyPhen-2 (http://genetics.bwh.harvard.edu/pph/), PMUT (http://mmb2.pcb.ub.es:8080/PMut/), and PANTHER (http://www.pantherdb.org/tools/csnpScoreForm.jsp).

### Statistical analysis

Continuous data were presented as the mean ± standard deviation (SD), and the differences between the two groups were evaluated using one-way ANOVA analyses. Frequencies were calculated as the proportion of mutation carriers among all participants. The differences in the overall frequencies of *BRCA1* and *BRCA2* mutations between groups were evaluated using Chi-square tests and Fisher’s exact tests. The statistics were performed using SPSS version 17.0 software for Windows.

## Results

### Patient features

A total of 133 unrelated patients with personal and family histories of breast and/or ovarian cancer underwent *BRCA1* and *BRCA2* germline mutation screening. All of the patients were from the Zhejiang province in eastern China. In our cohort of 133 breast cancer families, there were 2.3 ± 0.7 (mean number ± SD) occurrences of breast cancer per family. The age of breast cancer onset ranged from 22 years to 74 years. The mean age at diagnosis was 43.0 ± 9.3 (mean age ± SD) years. Ovarian cancer was present in 12.0 % (16/133) of all families.

### *BRCA1* deleterious mutations

In this cohort of 133 familial breast/ovarian cancer patients, 13 deleterious mutations in *BRCA1* were found in 18 unrelated patients, including five mutations that were reported in our previous study [13] (Table 1). None of the mutations had been registered in the 1000 Genomes database. The majority of the mutations were either nonsense or frameshift mutations with the exception of c.5467 + 1G > A and c.5468-1del8. Six mutations (46.2 %) were located in exon 11, and others were located in exon 19, exon 20, intron 23 and exon 24. There were five novel deleterious mutations (c.3295delC, c.3780_3781delAG, c.4063_4066delAATC, c.5161C > T and c.5173insA) that had not been registered in the BIC or any other public database. Moreover, two of the mutations (c.5468-1del8 and c.1465G > T) had only been previously reported in Chinese population. In this cohort, we detected four recurrent mutations (c.3780_3781delAG, c.5154G > A, c.5468-1del8 and c.5470_5477del8), which accounted for 30.8 % (4/13) of the total mutations. The mutation c.5470_5477del8 occurred three times, and the others occurred twice. The mean age at diagnosis of these *BRCA1* mutation carriers was 39.9 ± 8.1 (mean age ± SD) years (Table 2). No significant differences in the mean age at diagnosis between the *BRCA1* mutation carriers, *BRCA2* mutation carriers and non-carriers were found.

**Table 1:** *BRCA1* and *BRCA2* deleterious germline mutations in 133 Chinese women with familial breast/ovarian cancer

Gene	No. of patient	Exon	Systematic nomenclature	BIC nomenclature	Amino acid change	References
*BRCA1*	1	11	c.1465G > T	1584G > T	E489X	Zhi et al. [7]
	1	11	c.1945G > T	2064G > T	E649X	BIC
	1	11	c.3295delC	3414delC	P1099LfsX10	Novel
	2	11	c.3780_3781delAG	3899_3900delAG	L1260FfsX6	Novel
	1	11	c.4063_4066delAATC	4182_4185delAATC	N1355KfsX10	Novel
	1	11	c.4065_4068delTCAA	4184_4187delTCAA	N1355KfsX10	BIC
	2	19	c.5154G > A	5273G > A	W1718X	BIC
	1	19	c.5161C > T	5280C > T	Q1721X	Novel
	1	19	c.5173insA	5292insA	E1725EfsX7	Novel
	1	20	c.5251C > T	5370C > T	R1751X	BIC
	1	Intron23	c.5467 + 1G > A	IVS23 + 1G > A	Splicing defect	BIC
	2	Intron23	c.5468-1del8	5587-1del8	Splicing defect	Zhang et al. [11]
	3	24	c.5470_5477del8	5589_5596del8	I1824DfsX3	BIC
*BRCA2*	1	Intron1	c.1-40delGA	IVS1-1deGA	Splicing defect	Novel
	1	5	c.469_473delAAGTC	697_701delAAGTC	K157SfsX24	Novel
	1	9	c.755_758delACAG	983_986delACAG	T251XfsX1	BIC
	2	11	c.3109C > T	3337C > T	Q1037X	BIC
	1	11	c.4487delC	4715delC	P1496QfsX8	Novel
	1	11	c.5495delC	5723delC	S1832LfsX8	Novel
	3	11	c.5682C > G	5910C > G	Y1894X	BIC
	1	11	c.6141 T > A	6369 T > A	Y2047X	Novel
	1	11	c.6359C > G	6587C > G	S2120X	Novel
	1	15	c.7588C > T	7816C > T	Q2530X	Novel

*BIC* Breast Cancer Information Core

**Table 2:** Mean age at diagnosis in different *BRCA1* and *BRCA2* status

	*BRCA1*	*BRCA2*	Non-carriers	*P* ^a^	*P* ^b^	*P* ^c^
Number	18	13	102			
Mean age (±SD)	39.9 (±8.1)	41.1 (±6.5)	43.9 (±9.7)	0.74	0.11	0.31

*SD* standard deviation

^a^ *BRCA1* compare to *BRCA2* mutation carriers

^b^ *BRCA1* mutation carriers compare to non-carriers

^c^ *BRCA2* mutation carriers compare to non-carriers

### *BRCA2* deleterious mutations

A total of 10 deleterious mutations in *BRCA2* were found in 13 familial breast/ovarian cancer patients in this cohort (Table 1). None of these mutations had been registered in the 1000 Genomes database. The mean age at diagnosis of these *BRCA2* mutation carriers was 41.1 ± 6.5 (mean age ± SD) years (Table 2). Nine mutations were either nonsense or frameshift mutation, and the remaining mutation c.1-40delGA, which resulted in the deletion of a guanine in intron 1 and an adenine in exon 2, was a splicing site mutation. Sixty percent (6/10) of the all of the mutations were located in exon 11. There were seven novel mutations (c.1-40delGA, c.4487delC, c. 469_473delAAGTC, c.5495delC, c.6141 T > A, c.6359C > G and c.7588C > T) in this cohort, and these mutations represented 70 % (7/10) of the mutations in this gene. Two recurrent mutations (c.3109C > T and c.5682C > G) were detected in this cohort, and both of them were registered in the BIC.

### Frequencies of *BRCA1* and *BRCA2* deleterious mutations

A total of 23 deleterious mutations of *BRCA1* and *BRCA2* were identified in 31 familial breast/ovarian cancer patients, and the frequency was 23.3 % (31/133; Table 3). The frequencies of *BRCA1* and *BRCA2* mutations were 13.5 % (18/133) and 9.8 % (13/133), respectively.

**Table 3:** Frequencies of *BRCA1* and *BRCA2* germline deleterious mutations in different groups of patients

Features	Number of total cases	*BRCA1* mutation (%)	*BRCA2* mutation (%)	Overall mutation (%)	*P*-value
Total	133	18 (13.5)	13 (9.8)	31 (23.3)	
Age at onset					
≤40 years	51	11 (21.6)	6 (11.8)	17 (33.3)	0.031
>40 years	82	7 (8.5)	7 (8.5)	14 (17.1)	
Number of breast cancer cases in a family					
≤2	99	12 (12.1)	8 (8.1)	20 (20.2)	0.148
>2	34	6 (17.6)	5 (14.7)	11 (32.4)	
With a family history of ovarian cancer					
Yes	16	6 (37.5)	2 (12.5)	8 (50.0)	0.012
No	117	12 (10.3)	11 (9.4)	23 (19.7)	
Bilateral breast cancer					
Yes	15	3 (20)	3 (20)	6 (40)	0.115
No	118	15 (12.7)	10 (8.5)	25 (21.2)	

In the subgroup analysis, the highest overall *BRCA1* and *BRCA2* mutations rate was 50.0 % (8/16) in the breast cancer patients with family histories of ovarian cancer. The overall mutation rate of the two genes in the patients who were diagnosed at or before the age of 40 was higher than that of the counterpart group. Compared with the breast cancer patients with fewer than two relatives affected by breast cancer or unilateral breast cancer, the overall mutation rates were higher in the patients with two or more relatives affected by breast cancer or bilateral breast cancer, but these differences did not reach statistical significance (*P* = 0.148 and *P* = 0.115, respectively).

### Haplotype analysis of recurrent mutations

Four recurrent *BRCA1* mutations (c.3780_3781delAG, c.5154G > A, c.5468-1del8 and c.5470_5477del8) and two recurrent *BRCA2* mutations (c.3109C > T and c.5682C > G) were identified in unrelated breast cancer patients. As haplotype analysis of *BRCA1* c.5470_5477del8 mutation and *BRCA2* c.3109C > T mutation had been performed in Chinese high risk breast cancer patients [9, 10, 12], we performed haplotype analysis on the other four recurrent mutations in this study. Our results showed that carriers with the recurrent *BRCA1* c.5154G > A mutation shared the same haplotype, as well as carriers with the recurrent *BRCA1* c.5468-1del8 mutation, which suggested that these two putative founder mutations were derived from a common ancestor (Table 4). The three carriers with *BRCA2* c.5682C > G mutation sharing only two alleles (D13S171 and D13S1698) out of eight alleles implied that they might be not derived from a common ancestor (Table 5).

**Table 4:** Haplotype analysis of *BRCA1* recurrent mutations carriers

Mutation	Patient No.	D17S855	D17S1322	D17S1323	D17S1326	D17S1327
c.3780_3781delAG	1	145/147	113/116	150/152	108/110	128/130
	2	141/143	119/122	156/160	86/88	158/160
c.5154G > A	3	**143**/141	**122**/119	**156**/156	**90**/88	**154**/152
	4	**143**/151	**122**/116	**156**/152	**90**/104	**154**/130
c.5468-1del8	5	**147**/141	**116**/122	**152**/146	**104**/106	**128**/130
	6	**147**/145	**116**/113	**152**/150	**104**/102	**128**/130

Shared haplotypes are bolded

**Table 5:** Haplotype analysis of *BRCA2* c.5682C > G mutation carriers

Patient No.	D13S171	D13S217	D13S267	D13S289	D13S1304	D13S1695	D13S1698	D13S1699
7	**224**/238	**164**/172	**144**/151	**146**/156	157/159	**211**/215	**158**/156	161/163
8	**224**/224	168/170	151/159	144/156	**153**/155	209/213	**158**/156	**157**/155
9	**224**/228	**164**/160	**144**/142	**146**/144	**153**/149	**211**/207	1**58**/160	**157**/159

Shared haplotypes are bolded

### UVs of *BRCA1* and *BRCA2*

In addition to deleterious mutations, we identified 11 UVs (seven in *BRCA1* and four in *BRCA2*; Table 6). Comparisons with the 1000 Genomes database revealed that only *BRCA1* c.2286A > T (R762S) had been reported in a Pakistani population, and the frequency of the T allele was 0.5 % in that population. None of the UVs had previously been found in the Chinese population. The majority of the variants were novel, with the exception of the mutation c.2286A > T in *BRCA1*, which is registered in the BIC, and c.2726A > T in *BRCA1*, which was recently reported in a Chinese population previously [8]. The possibility that each of the UVs would disrupt the function of *BRCA1* or *BRCA2* was predicted in silico, and the results varied according to the different algorithms used.

**Table 6:** *BRCA1* and *BRCA2* germline UVs in 133 Chinese women with familial breast/ovarian cancer

Gene	No. of patient	Exon	Systematic nomenclature	BIC nomenclature	Amino acid change	References	Align-GVGD	SIFT	PROVEAN	PolyPhen-2	PMUT	PANTHER
*BRCA1*	1	11	c.1679A > T	1798A > T	D560V	Novel	C0	Damaging	Deleterious	Possibly damaging	Neutral	Deleterious
	1	11	c.1537C > G	1656C > G	H513D	novel	C0	Tolerated	Deleterious	Benign	Pathological	Neutral
	1	11	c.2286A > T	2405A > T	R762S	BIC	C0	Damaging	Deleterious	Benign	Pathological	Neutral
	1	14	c.4445A > C	4564A > C	D1482A	novel	C0	Damaging	Neutral	Benign	Pathological	Neutral
	1	11	c.1966A > T	2085A > T	N656Y	novel	C0	Damaging	Deleterious	Possibly damaging	Neutral	Deleterious
	1	11	c.2340G > T	2459G > T	Q780H	novel	C0	Damaging	Deleterious	Probably damaging	Neutral	Deleterious
	1	11	c.2726A > T	2845A > T	N909I	BIC, Thirthagiri et al. [8]	C0	Damaging	Deleterious	Possibly damaging	Neutral	Neutral
*BRCA2*	1	10	c.1568A > G	1796A > G	H523R	novel	C0	Damaging	Neutral	Benign	Pathological	Neutral
	1	11	c.3904A > G	4132A > G	T1302A	novel	C0	Tolerated	Deleterious	Benign	Neutral	Neutral
	1	11	c.5590G > A	5818G > A	D1864N	novel	C0	Damaging	Neutral	Benign	Neutral	Neutral
	1	11	c.6763A > T	6991A > T	T2255S	novel	C0	Damaging	Neutral	Possibly damaging	Neutral	Deleterious

## Discussion

*BRCA1* and *BRCA2* are the most important genetic susceptibility genes for breast/ovarian cancer in both Caucasian and Chinese populations. The spectrum and frequencies of mutations in these two genes in Chinese women with familial breast/ovarian cancer have been insufficiently explored to date. Moreover, the penetrance has not yet been investigated. Due to the limited knowledge on hereditary breast/ovarian cancer, there is no genetic counseling or testing services available in Mainland China.

Our results demonstrated that the frequency of *BRCA1* and *BRCA2* mutations among Chinese women with familial breast/ovarian cancer was 23.3 %. Similar results have been reported in the Korean population [23], Hispanic population [24] and Africa American population [25]. However, the frequency observed in the current study is lower than that reported in an Ashkenazi Jewish population, in which the frequency of *BRCA1* and *BRCA2* mutations was 69 % [25]. Compared with other reports about Chinese populations, the frequency found in our cohort was the highest in patients with familial breast/ovarian cancer. Li et al. [9] used PCR-DHPLC assay to screen for *BRCA1* and *BRCA2* mutations in 241 women with familial breast cancer from northern or southern China and found a frequency of 12.9 %. Although the PCR-DHPLC assay is cost-effective for screening for genetic mutations, a considerable number of disease-associated mutations may have been missed by this indirect detection method [26]. Zhang et al. [11] reported that the frequency of *BRCA1* and *BRCA2* mutations in northern Chinese familial breast cancer patients was 10.5 % (43/409) based on PCR-sequencing assay. The enrolment criteria and mutation detecting assay used in this were comparable with the criteria used in our study, but the reported frequency was much lower than that observed in the present study. In their subgroup analysis, the highest frequency was 23 % in the patients whose tumors had been diagnosed at or before the age of 40 years. However, the frequency reached 33.3 % in this group of patients in our cohort. Moreover, in the study conducted by Kwong et al., [12] the frequency of *BRCA1* and *BRCA2* mutations in high-risk breast/ovarian cancer patients was 15.3 % (69/651). These authors also employed the conventional PCR-sequencing assay, and the patients were recruited from southern China. The proportion of high-risk breast/ovarian cancer patients, including familial breast cancer patients and early-onset cases and the frequency of two-gene mutations were much lower in the early-onset patients than in the familial breast cancer cases. Large genomic rearrangements account for 4–28 % of all *BRCA1* and *BRCA2* mutations [27], and such mutations have been found in Chinese women at a high risk for breast cancer [28–32]. Because the PCR-sequencing assay cannot detect these rearrangements, the frequency of mutations in our cohort might have been underestimated, and the frequency of *BRCA1* and *BRCA2* mutations in the eastern Chinese population could be significant.

Although several studies have reported that the *BRCA2* mutations are more frequent than *BRCA1* mutations in Asian population [11, 12, 33, 34], *BRCA1* mutations seemed to be more prevalent in our cohort. This finding might be attributable to two points. First, most studies have reported that *BRCA2* mutations predominantly occur in relatively late-onset breast cancer patients compared with *BRCA1* mutations [11, 35], but the patients enrolled in our study were much younger than those in other studies, which might have resulted in an underestimation of the contribution of *BRCA2* mutations. Second, a greater number of recurrent mutations were found in *BRCA1* than in *BRCA2* in our study, which elevated the frequency of *BRCA1* mutations.

In the present study, we found that 52.2 % (12/23) of the deleterious mutations were novel; these mutations included five mutations in *BRCA1* and seven mutations in *BRCA2*. In our previous systemic analysis of the spectrum of *BRCA1* and *BRCA2* mutations in Han Chinese women, we reported that 56.3 % (40/71) and 47.9 % (35/73) of the *BRCA1* and *BRCA2* mutations were novel, respectively [22]. It seems that the spectrum of *BRCA1* and *BRCA2* mutations in Chinese women exhibit unique features. The *BRCA2* mutation c.1-40delGA in our cohort was novel. Bakker et al. [36] found a *BRCA2* c.1-40 G > A mutation in a Japanese Fanconi anemia family. The functional analysis of these authors used a mouse embryonic stem cell-based assay that revealed that this mutation caused aberrant splicing, reduced transcript levels and hypersensitivity to DNA damaging agents, suggesting that this mutation was likely pathogenic. These authors thought that this finding was relevant for mutation analysis in hereditary breast and ovarian cancer syndrome families in a diagnostic setting. The mutation c.1-40delGA, which deletes a guanine in intron 1 and an adenine in exon 2 and causes the loss of the donor site of intron 1, should also be pathogenic.

Six *BRCA1* and *BRCA2* recurrent mutations were identified in multiple patients, and these accounted for 45.2 % (14/31) of the total patients with mutations. Of these mutations, one (c.3780_3781delAG) was novel, another (c.5468-1del8) was recently reported in Chinese women [11], and the remaining four had been reported in the BIC database. Founder mutations provide population-specific genetic risk assessment, and facilitate genetic mutation screening. Thus far, few studies have suggested that putative founder mutations of *BRCA1* and *BRCA2* might exist in Chinese women at a high risk for breast cancer, such as the c.981delAT and c.5470_5477del8 mutations in *BRCA1* and the c.3109C > T, c.7436_7805del370 and c.9097_9098insA mutations in *BRCA2* [9, 10, 12]. In our cohort, the *BRCA1* c.5470_5477del8 mutation and *BRCA2* c.3109C > T mutation were both recurrent, but no other three putative founder mutations was found. Our haplotype analysis revealed that *BRCA1* c.5154G > A and c.5468-1del8 mutations were the two putative founder mutations. Since there are only two patients reported for each of the putative founder mutation, the founder effects are needed to be investigated by larger sample size of patients. In our previous study, we reported that the most common recurrent mutations in Chinese women at high risk for breast cancer are c.5470_5477del8 in *BRCA1* and c.3109C > T in *BRCA2* [22], which were reported to be the putative founder mutations. However, the study that enrolled the greatest number of familial breast cancer patients from northern China did not find these six putative founder mutations except the *BRCA1* c.5468-1del8 mutation [11]. The discrepancy regarding the founder mutations in Chinese familial breast cancer patients may be due to geographic differences. The characterization of *BRCA1* and *BRCA2* founder mutations and association between the founder mutations and breast cancer risk should be studied in a large-scale Chinese population size.

Although, elevated mutation rates of *BRCA1* and *BRCA2* were found in patients who had been diagnosed at or before 40 years of age, no significant differences were found between the *BRCA1* mutation carriers, *BRCA2* mutation carriers and non-carriers when compared to a mean age at diagnosis. The inconsistent results implied that these observations did not withstand multiple comparisons in our cohort. Breast cancer patients with family histories of ovarian cancer exhibited the highest overall mutation rate of *BRCA1* and *BRCA2*, which implied that *BRCA1* and *BRCA2* mutations are more likely to occur in families with a history of both breast and ovarian cancer. This result is consistent with those of other studies [9, 11].

Eleven UVs were found in our study, and the potentials for these variants to disrupt the functions of *BRCA1* and *BRCA2* varied according to the algorithm program used. The UVs accounted for nearly 1/3 of the total mutations/variants in this study. The risks of breast and ovarian cancer in the UVs carriers might be as high as those in the carriers of the classical pathogenic mutations. A variety of approaches have been used to investigate the clinical relevance of these UVs. Co-segregation analysis is regarded as a robust approach because it is directly related to the disease risk and is not affected by selection bias [37]. The absence of co-segregation provides strong evidence against pathogenicity. Unfortunately, the samples required for us to perform co-segregation analysis of UVs and the deleterious mutations in the multi-tumor families were not available.

## Conclusions

In the present study, we found that the frequency of *BRCA1* and *BRCA2* mutations was 23.3 % in our cohort of 133 Chinese women with familial breast/ovarian cancer, and the frequency of *BRCA1* and *BRCA2* mutations was 50 % in patients with a familial history of both breast cancer and ovarian cancer. The spectrum of *BRCA1* and *BRCA2* mutations in the Chinese population are quite different from those in other ethnicities. Six recurrent mutations were detected in this study, in which two recurrent *BRCA1* mutations were identified as putative founder mutations, and a larger sample size is required to determine the founder effects of these two mutations in Chinese women. *BRCA1* and *BRCA2* mutations account for a considerable proportion of Chinese hereditary breast/ovarian cancer patients, and the penetrance of these two genes should be investigated because such investigations will be very important for the development of a preventive treatment strategy in China.

## Abbreviations

BIC: Breast cancer information core
PCR: Polymerase chain reaction
SD: Standard deviation
UVs: Unclassified variants
